# Abundance and Dynamic Distribution of Antibiotic Resistance Genes in the Environment Surrounding a Veterinary Antibiotic Manufacturing Site

**DOI:** 10.3390/antibiotics10111361

**Published:** 2021-11-08

**Authors:** Junjie Miao, Zhendong Yin, Yuqin Yang, Yiwen Liang, Xiangdong Xu, Hongmei Shi

**Affiliations:** Key Laboratory of Environment and Human Health of Hebei Province, School of Public Health, Hebei Medical University, Shijiazhuang 050017, China; miao_4820@163.com (J.M.); zhendongyin123@163.com (Z.Y.); yuqinyang78@163.com (Y.Y.); liangyiwen668@163.com (Y.L.); xuxd@hebmu.edu.cn (X.X.)

**Keywords:** antibiotic resistance genes, environmental media, abundance, dynamic distribution, qPCR

## Abstract

**Background:** Antibiotics releasing from the manufacturing sites to the surrounding environment has been identified as a risk factor for the development of antibiotic resistance of bacterial pathogens. However, the knowledge of the abundance and distribution of antibiotic resistance genes (ARGs) influenced by antibiotic pollution is still limited. **Methods:** In this work, the contamination by resistance genes of the environmental media including an urban river and soil along the river located near the sewage outlet of a veterinary antibiotic manufacturing site in Shijiazhuang, China, was assessed. The abundance and dynamic distribution of ARGs in different sampling points and during different seasons were analyzed using fluorescent quantitative PCR method (qPCR). **Results:** A total of 11 resistance genes, one integron and one transposon were detected in water and soils around the pharmaceutical factory, and among which, the sulfonamide resistance genes *sul1* and β-lactam resistance genes *blaSHV* were the most abundant genes. The relative abundance of ARGs in both river water and soil samples collected at the downstream of the sewage outlet was higher than that of samples collected at the upstream, non-polluted areas (*p* < 0.05). The mobile genetic elements (MGEs) integron in river was significantly correlated (*p* < 0.05) with the relative abundance of ARGs. **Conclusions:** The results indicate that the discharge of waste from antibiotic manufacturing site may pose a risk of horizontal transfer of ARGs.

## 1. Introduction

Antibiotics are widely used to prevent and treat bacterial infectious diseases, and to promote the growth and increase the feed efficiency in animal [1]. Most veterinary antibiotics are not completely absorbed in the animal intestines, but are excreted into the environment through feces and urine in the form of prototypes [2]. In recent years, the overuse, abuse, and unmonitored discharge of antibiotics have promoted the occurrence and spread of antibiotic resistance genes (ARGs) and antibiotic-resistant bacteria [3,4,5]. ARGs could advance bacteria “along the path to becoming superbugs”. As emerging pollutants, the spread and diffusion of resistance genes in the environment pose a serious threat to public health. Infections caused by antibiotic-resistant bacteria in the European Community/EU region have been very significant since 2007, and more than thirty thousand people have died each year due to infections caused by different resistant bacteria [6]. According to the US Centers for disease control, at least two million Americans are infected with bacteria that cannot be treated with antibiotics every year, and at least 23,000 of them have died. World Health Organization (WHO) predicted that the spread of antibiotic resistance would kill 300 million people in 2050. In addition, according to the latest research results, drug-resistant strains not only exist in patients, but also in healthy people who may carry drug-resistant genes, which is a public health problem that is easy to be ignored to deal with drug-resistant bacteria [7,8]. WHO released the “Draft global action plan on antimicrobial resistance” in 2014, and pointed out that drug resistance is one of the most urgent public health problems in the world. China also issued the national action plan to curb bacterial drug resistance in 2016, which aims to strengthen the scientific management of antibiotics, curb the development and spread of bacterial drug resistance, and maintain the health of the people. ARGs have become a widespread public health and concern [9,10,11].

A large number of bulk antibiotic drugs and their preparations are produced every year. Sewage discharged from an antibiotic production process is treated before being discharged into the environment. Studies have shown that the current wastewater treatment process cannot completely eliminate antibiotics and antibiotic resistance genes, causing some residues amounts to still be discharged into the environment [12,13,14,15]. Antibiotic residues in the environment exert selective pressure on bacteria even at low concentrations, and possibly induce the generation of ARGs, promoting their horizontal transfer, and accelerating their dissemination [16,17]. Residual antibiotics and ARGs can enter into drinking water systems, which in turn increase the possibility of antibiotic resistance dissemination among the population [18]. In addition, antibiotics and ARGs will be transferred to soils through crop irrigation [19], composting of animal feces [20] etc., thus entering the food chain, posing threats to human health [21,22,23].

China is a major producer and consumer of antibiotics. It produces nearly 300,000 tons of antibiotic raw materials every year, of which more than 80,000 tons are used in animal husbandry and aquaculture [18,24]. In recent years, industrial discharges from antibiotic manufacturing have been recognized as a risk factor fostering ARGs development and dissemination [25]. ARGs have been detected in various environmental media. Liu et al. [26] have investigated the relative abundance of sulfonamide resistance genes in waters in Beijing area, and found to be as high as 7.11 × 10^−2^ to 1.18 × 10^−1^. The follow-up study by Chen et al. [27] also showed that most of the 27 resistance genes found in Chaobai River sediments were abundant and positively correlated with integrons. The pollution of resistance genes in groundwater environment has been studied by Szekeres et al. [28]. In their results, at different locations of an urban area, the relative abundances of 11 resistance genes has been found to range from 6.61 × 10^−7^ to 2.30 × 10^−1^, and the diversity of species in the contaminated groundwater has also been found to increase significantly. Xie et al. and Gaviria-Figueroa et al. [29,30] have detected a variety of ARGs in air around a sewage treatment factory, and confirmed that the bioaerosol in the air could promote the diffusion and spread of bacteria and drug-resistant genes. Many studies have shown that horizontal gene transfer (HGT) is the main mechanism for the widespread distribution of bacterial drug resistance [31,32]. The combination of ARGs and mobile genetic elements (MGEs) can lead to horizontal transfer in the environment, thereby can accelerate the dissemination of ARGs and increase the possibility of drug-resistant contamination of bacteria [33].

Shijiazhuang is close to the capital Beijing. It is an antibiotic production base of China. Antibiotics are the main products of some pharmaceutical groups, such as the North China Pharmaceutical group and the Shijiazhuang Pharmaceutical Group. A large number of antibiotic drugs are produced and distributed at Shijiazhuang. The total output of antibiotic raw materials from the North China Pharmaceutical Group accounts for about 15% of the country’s total output, and its production of penicillin, semi-synthetic penicillin, and other intermediates rank first in Asia. A veterinary drug manufacturer located in the urban area has been producing a variety of veterinary antibiotics, including sulfonamide, tetracycline, and beta-lactam antibiotics, for more than 20 years. It has also been discharging its effluent into the nearby Min-xin river. The long-term effect of low-dose residue in water discharged from antibiotic manufacturing sites on the surrounding environment has been identified as a risk factor for the development of antibiotic resistance of bacterial pathogens [25,34]. The aims of the present study are: (i) To detect the types of resistance genes in the water of Min-xin river and soil from different sampling points around this veterinary drug production site; (ii) to analyze the abundance and dynamic distribution of ARGs during different seasons; and (iii) to evaluate the pollution of ARGs caused by antibiotic-manufacturing discharge in the surrounding environmental media. 

## 2. Results

### 2.1. Detection of Resistance Genes by qPCR Assay

**Figure 1:**
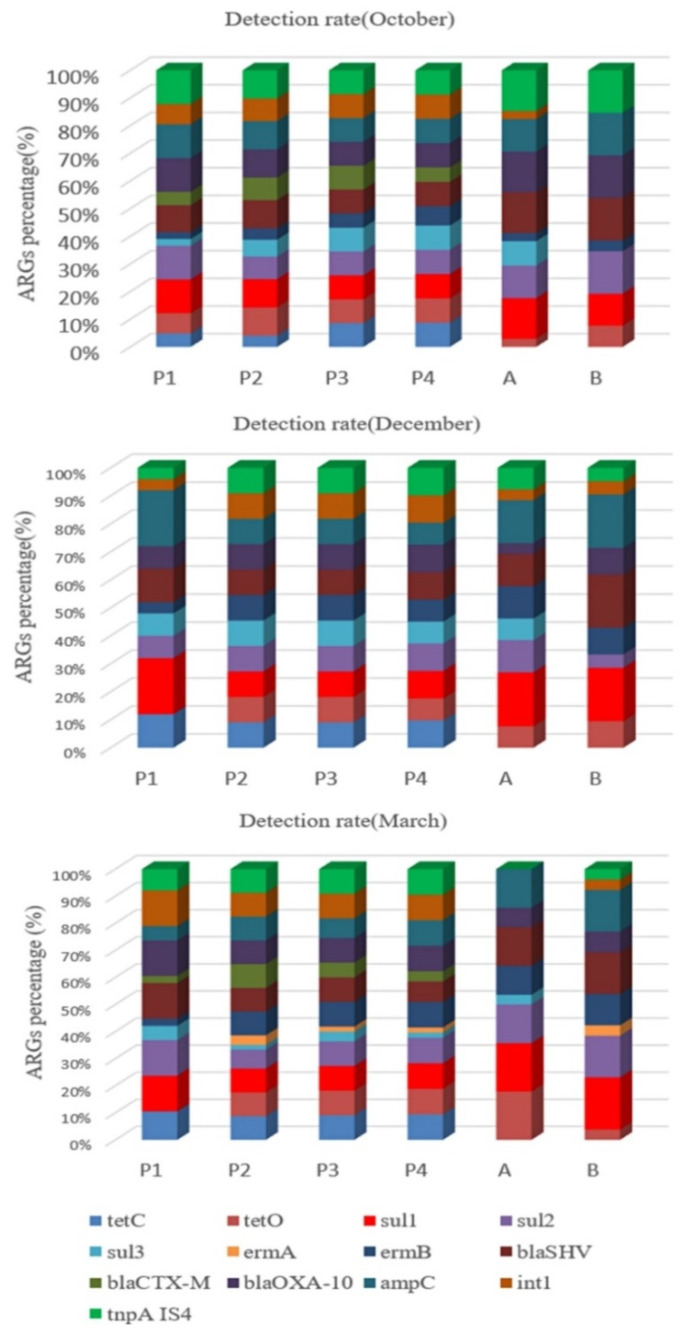
Detection rate of resistance genes in samples collected in different months.

A total of 11 resistance genes (*tetC, tetO, sul1, sul2, sul3, ermA, ermB, blaOXA-10, blaSHV, blaCTX-M, ampC*), class 1 integrons *intI,* and one transposon *tnpA-IS4* were detected in the collected water and soil samples by q-PCR technique, as shown in Figure 1.

As can be seen from Figure 1, the frequency of detection of these genes varied greatly. For samples collected in October, the sulfonamide resistance genes *sul1*, the β-lactam resistance genes *blaOXA-10* and *ampC*, and transposon *tnpA-IS4* had the highest detection rates in water samples, and β-lactam resistance genes *blaSHV*, *blaOXA-10* and transposon *tnpA-IS4* had the highest detection rates in soil samples. The detection rate of sulfonamides resistance gene *sul1* was highest in both water and soil samples collected in December. For samples collected in March, the detection rate of sulfonamides-resistant gene *sul1*, β-lactam-resistant gene *blaOXA-10* and transposon *tnpA-IS4* was highest in water samples, and that of resistance gene *sul1* was also highest in soil samples. Although the macrolide resistance gene *ermA* was also detected, its detection rate was the lowest. 

### 2.2. Analysis of Absolute Abundance of ARGs by qPCR 

**Figure 2:**
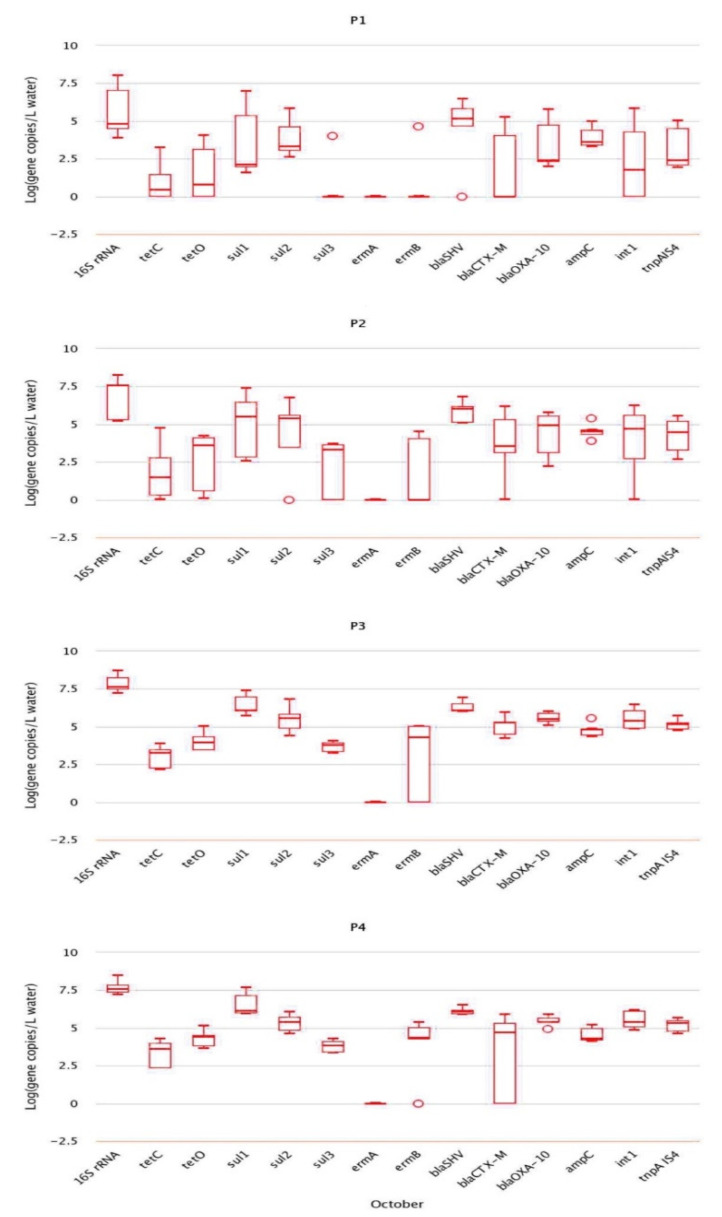
Abundance of resistance genes in samples collected in October.

The absolute abundance of genes is the actual concentration obtained by converting the copy number of target genes in the samples obtained by qPCR according to the dilution multiple. Unit of absolute abundance is copies/g (dry mass of soil) or copies/L (water sample). Resistance genes with the highest absolute abundance in water and soil samples collected in October included sulfonamide resistance gene *sul1* (4.39 × 10^7^ copies/L), followed by β-lactam resistance gene *blaSHV* (2.73 × 10^6^ copies/g) (Figure 2). The resistance of genes in water samples from different sampling points was not significantly different. However, the absolute abundance of sulfonamide resistance gene *sul1*, β-lactam resistance gene *blaOXA-10*, and transposon *tnpA-IS4* were significantly different (*p* < 0.05), and the absolute abundance at P3 and P4 was significantly higher than that at P1. In soil samples, the absolute abundance of resistance genes at points A and B was not obviously different except for that of transposon *tnpA-IS4* (*p* < 0.05). Additionally, the absolute abundance of tnpA-IS4 at point B was higher than that at point A.

**Figure 3:**
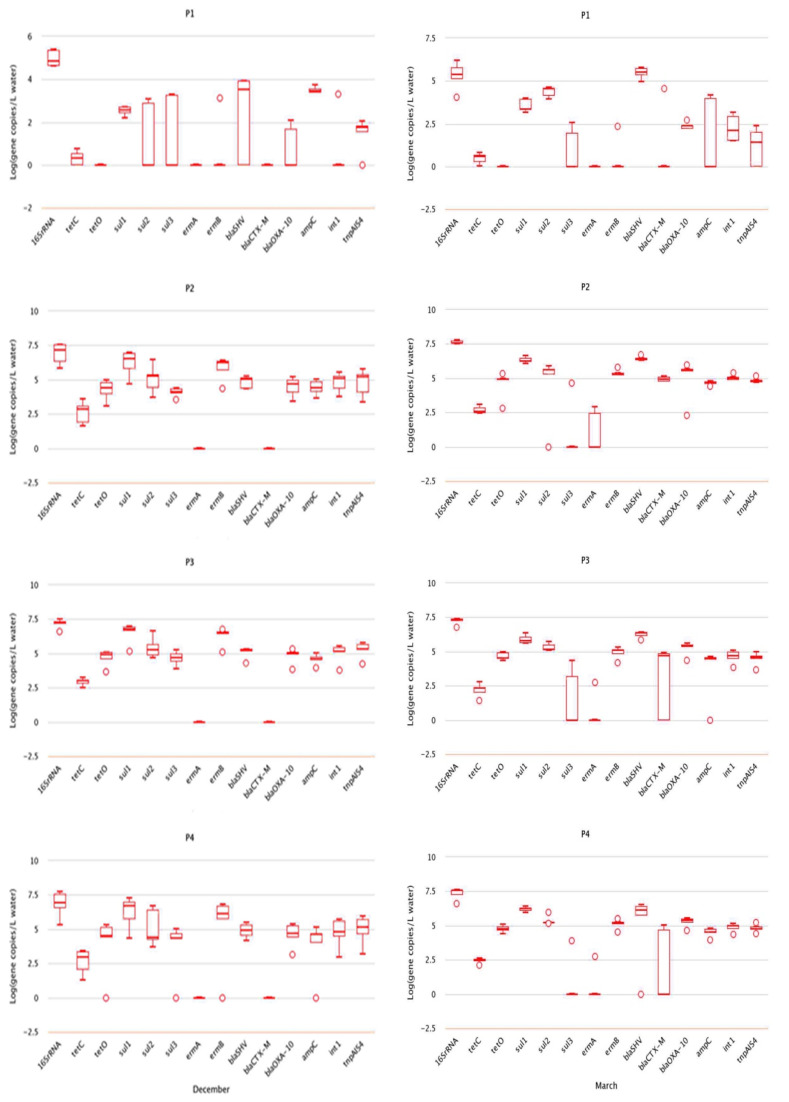
Abundance of resistance genes in samples collected in December and March.

In the water and soil samples collected in December, resistance genes with the highest absolute abundance in water was sulfonamide resistance gene *sul1* (1.79 × 107 copies/L), while that in soils was *sul2* gene (2.53 × 108 copies/g) (Figure 3). The absolute abundance of resistance genes *tetC, sul1, sul2, sul3, blaOXA-10, blaSHV, ampC*, and *tnpA-IS4* in water from different sampling points were significantly different (*p* < 0.05), and the absolute abundance in points P2, P3, and P4 was obviously higher than in point P1.

The absolute abundance of β-lactam resistance gene *blaSHV* was highest both in water samples (5.12 × 10^6^ copies/L) and soil samples (4.17 × 10^6^ copies/g) collected in March. The absolute abundance of *tetC*, *sul1*, *sul2*, *blaSHV*, *blaOXA-10*, *intI* and *tnpA-IS4* in water samples from different sites was significantly different (*p* < 0.05), and the absolute abundance in points P2, P3, and P4 was also obviously higher than that in point P1, as shown in Figure 3.

### 2.3. Relative Abundance of ARGs

The total amounts of microorganisms in water and soil samples were different, as a result, the total amount of resistance genes was different. Therefore, the concentration of ARGs in the tested sample was normalized to that of the internal reference gene 16S rRNA to obtain the relative abundance of ARGs, which is equal to the absolute abundance of target genes/the absolute abundance of 16S rRNA genes. The calculation of relative abundance means that the influence of microorganisms in the environment is excluded. Table 1 lists the results from analysis of variance between resistance genes in sampling points at different times. Among all genes, only *sul2* and *blaSHV* genes in water samples collected in March had the relative abundance greater than 0. In the abovementioned results in Section 2.2, the absolute abundance of *blaSHV* in both water and soil samples collected in March was the highest. The results of relative abundance were consistent with those of the absolute abundance. One-way analysis of variance showed that the relative abundance of most resistance genes detected in samples collected from different sampling points was significantly different. The relative abundance of β-lactam resistance gene *ampC* in water samples collected in October at different sampling points was significantly different (*p* < 0.05), and its relative abundance at P3 and P4 was significantly higher than that at P1.

The relative abundance of resistance genes *sul1*, *blaOXA-10*, and *ampC* in water samples collected in December at different sampling points was also significantly different (*p* < 0.05), and the relative abundance at points P2, P3, and P4 was significantly higher than at point P1. The relative abundance of transposon *tnpA-IS4* in samples collected from different sampling points was significantly different, and the relative abundance at points P2, P3, and P4 was also significantly higher than that at point P1. In the water samples collected in March, the relative abundance of *sul2*, *blaSHV,* and *ampC* resistance genes was significantly different (*p* < 0.05), and that at points P2, P3, and P4 was significantly higher than that at P1. The relative abundance of type I integron *intI* at different sampling points was also significantly different (*p* < 0.05), and its relative abundance at point P4 was obviously higher than that at point P1. All of the analysis results are listed in Table 1.

### 2.4. Correlation Analysis of ARGs with Integron intI and Transposon tnpA-IS4

The distribution and diffusion of resistance genes in the environment are usually affected by antibiotic selection pressure. The same selection pressure may simultaneously induce multiple resistance genes. Therefore, analysis of the correlation between ARGs can help understand their synergistic relationship. The diffusion of ARGs in the environment is mainly through horizontal transfer, in which mobile genetic elements (MGEs) play a very important role [35]. The Pearson correlation analysis of the data showed that there was significant correlation between ARGs and the mobile genetic elements of *intI* and *tnpA-IS4* (see Table 2). For water samples collected in October, there was significant correlation between *tetC*, *tetO*, *sul1* resistance genes and integron *intI*. The correlation between *ermB, blaOXA-10,* and *ampC* resistance genes and transposon *tnpA-IS4* was significant.

For water samples collected in December, *tetO*, *ermB,* and *blaOXA-10* resistance genes were significantly correlated with integron *intI*. *Sul1*, *ermB*, *blaOXA-10* resistance genes were significantly correlated with transposon *tnpA-IS4*. 

For water samples collected in March, there was significant correlation between *sul1* and *ermB* resistance genes and the integron *intI*, as well as between *sul1*, *ampC,* and transposon *tnpA-IS4*.

### 2.5. Soil Moisture Content

In this experiment, the water content in soil samples collected at two sampling points A and B was determined. The results showed that in three out of five samples, the soil moisture content at point B was higher than that at point A. Soil moisture content data were listed in Table 3.

## 3. Discussion

The detection of antibiotic resistance gene in the Min-xin river water and soil near a veterinary antibiotics production site showed that sulfonamides antibiotic resistance gene *sul1* had the highest detection rate, indicating that this gene may be the dominant gene in this area. This is likely due to that sulfa antibiotics are more commonly used in this area, or it is possible that there is long-term exposure of sulfa drug residues. Similarly, Gao et al. [36] have shown that the prevalence, concentration, and detection rate of sulfa drug resistance genes in aquaculture environments were also the highest, and the most likely reason is that sulfa drugs are more commonly used. The results also showed that most ARGs detected in water samples collected from four points showed an increasing trend from P1 to P4. The detection rate of ARGs at point P1 was generally low, indicating that the pollution situation in this point is relatively low. In the water samples downstream of the sewage outlet, the detection rate was the highest, which is a proof that the sewage outlet, to a certain degree, is the source of pollution detected in the downstream river. Although the detection rate of ARGs in soil samples at point A and B was not high, it could still be seen that the detection rate of ARGs at point B was higher than that at point A. This is consistent with a study previously reported by Jahnavi et al. [37], which describes that the increase of detection rate of ARGs in river sediments, as compared with that in uncontaminated sediments, is influenced by the discharges.

The results from absolute abundance analysis results showed that most resistance genes detected at different sampling points were different, and the different months in which the samples were collected also had an impact on their abundance and changes of ARGs. The absolute abundance of ARGs at point P4 located downstream of the sewage outlet was higher than that at points P2 and P1 located at the upstream. This indicates that the sewage discharged from the sewage outlet has a certain promoting effect on the accumulation of ARGs in water in the river. Similar to this finding, a study by Kristiansson et al. [38] has demonstrated that a large amount of antibiotics that is discharged into the rivers can help in the enrichment of ARGs and *intl*, thereby can promote their spread. The diversity and composition of microbial communities in the environmental media can also be affected by temperature and change of seasons [39].

According to literatures, temperature is the main reason for the difference between results observed at different times. The temperature also changes with the change of seasons. Accordingly, the types and quantities of microorganisms in the water and soil change, and this can affect the horizontal transfer of ARGs [40]. Zheng et al. [41] have showed that the absolute abundance of ARGs in rivers around the city is highest in summer, and the change trend of the abundance is correlated with the local temperature.

**Figure 4:**
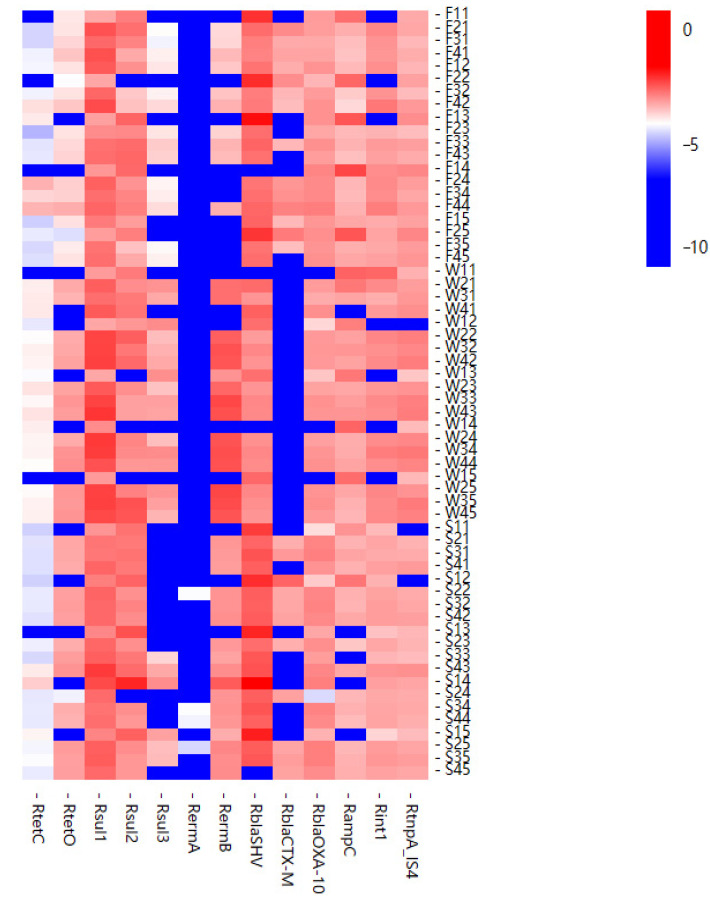
Heatmap showing relative abundance of ARGs in all samples.

A heat map of the relative abundance of resistance genes detected for all samples was prepared (Figure 4). As can be seen, the relative abundance of ARGs detected at different times was different. The results in all the three different months showed that sulfa *sul1* and β-*lactam blaSHV* were the main resistance genes. The relative abundance of tetracycline *tetC* was the lowest, and *ermA* was not detected the most times. The trend of relative abundance of ARGs in water samples was similar, and in most samples, the relative abundance at point P4 was significantly higher than that at point P1. That is, the relative abundance of ARGs downstream of the sewage outlet was higher than that of the non-polluted upstream area. This shows that the sewage discharged from the sewage outlet has an impact on the relative abundance of ARGs in the river, causing the relative abundance of integrons and transposons to increase correspondingly.

In this study, the detection rate of transposons in water and soil samples was relatively high, and a variety of ARGs showed significant correlation with integrons and transposons. Su et al. [42] have described that bacteria can share genetic information through the horizontal transfer of MGEs in the environment, and demonstrated that there is a transfer of ARGs between soil and rivers. Sewage discharge could significantly increase the transfer frequency of ARGs to *Escherichia coli* receptors. Chen et al. [43] have also reported significant correlation between *sul1*, *sul2*, and int1. Li et al. [44] and Yang et al. [45] have shown that there is significant correlation between *tetC* and int1. Zheng et al. [41] have shown that MGEs are the key factors driving the change of ARGs in rivers around cities. This indicates that the possibility for the horizontal transfer of ARGs is high, and mobile genetic elements *intI* and *tnpA-IS4* may promote the spread of antibiotic resistance genes.

Our previous studies [46,47] have shown that sulfonamides and β-lactams antibiotic residues were not detected in Min-xin river water and soil along the river [48]. This is likely due to that antibiotic drugs can be degraded in the environment. With the influence of temperature and pH of the environmental media, natural photosensitizers, etc., antibiotic drugs are prone to hydrolysis or photolysis [49]. One probable reason why antibiotic residues were not detected in the environmental media is that they had been degraded under the influence of environmental factors in the sampling sites. In addition, there is an influence from antibiotic selection pressure.

## 4. Materials and Methods

### 4.1. Reagents and Instrumentation

Reagents used in this study included genomic DNA extraction kit (Omega Bio-Tek, Norcross, CA, USA), SYBR green fluorescent dye (Quanshijin Technology Co., Ltd. Beijing, China) and primers, and plasmids (Shenggong Bioengineering Co., Ltd. Shanghai, China). Instrument used in the study included an electronic balance (Precision Scientific Instrument Co., Ltd. Shanghai, China), a Heal Force ultrapure water system (Likang Biomedical Technology Holding Group, Hong Kong, China), and a real-time fluorescent quantitative PCR machine (Bio-Rad CFX96, Hercules, CA, USA).

### 4.2. Sample Collection and Processing

**Figure 5:**
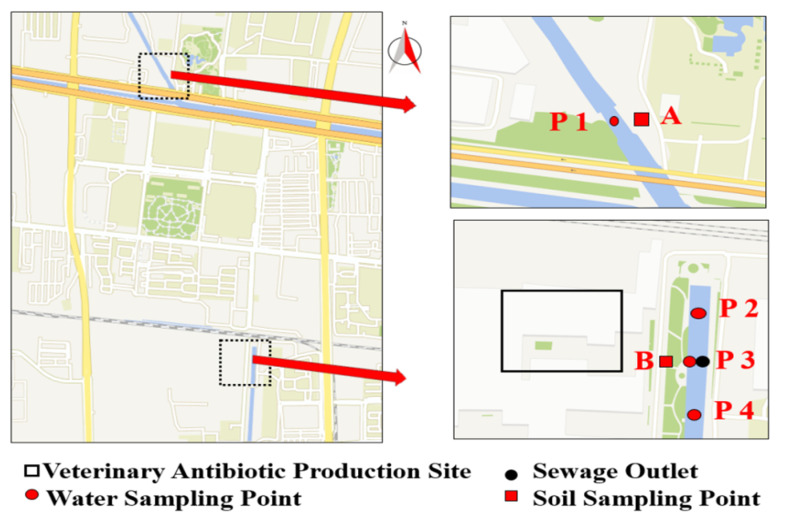
Geographical map of water and soil sampling sites.

River water and soil samples were sampled according to HJ/T 91-2002 and GB/T 36179-2018 of China National Standard, respectively. The sewage from the veterinary drug production factory was discharged to Min-xin River (Shijiazhuang, China). Water samples at the upstream and downstream of the sewage port were collected in October, December, and March of 2019, respectively. Soil samples were collected at various sampling points along the river at the same points where water samples were collected. Two soil sampling points (point A, point B) were set up to observe the impact of the wastewater generated by the veterinary antibiotics factory on the surrounding environment. Point B is located near the drug production site, and point A is located at the control point. Water samples were collected from four sampling points. As shown in Figure 5, point P1 is located at the upstream of the river, whereas point P2 is located at the midstream of the river, near the effluents of the pharmaceutical company. In addition, point P3 was the wastewater outlet of the pharmaceutical factory, while point P4 was at the downstream. 

Water sample from each sampling point was continuously collected for 5 days at different times, and a total of 1 L of water (at 0.5 m below the water surface) was collected in light-tight glass bottles. Using stainless steel sampling shovel, soil samples (at 10 cm below the soil surface) were collected and placed in a sterile collection bags. Water and soil samples after collection were all stored in a car refrigerator and then transported back by driving to the laboratory within two hours. Water sample was filtered through 0.22-μm polyethersulfone (PES) filters. The filter membrane was placed in a 50-mL centrifuge tube, and then stored at −20 °C. Soil samples were stored at −20 °C before processing. After measuring its moisture content, the collected soil was weighed to about 50 g and then transferred into a 50-mL centrifuge tube. The centrifuge tube was placed in a vacuum drying oven, in which the sample was dried to a constant weight.

### 4.3. Selection of 11 Resistance Genes

Eleven genes including *tetC*, *tetO*, *sul1*, *sul2*, *sul3*, *ermA*, *ermB*, *blaOXA-10*, *blaSHV*, *blaCTX-M*, *ampC*, class 1 integrons *intI* and one transposon *tnpA-IS4* were finally selected for detection in this work. First, we investigated the annual output and types of antibiotics produced by antibiotic manufacturers in this region, and then the possible pollution of antibiotic resistance genes in the environmental media in this area was analyzed. Then pre experiments were carried out to detect macrolides *ErmA/B/C*, quinolones *qnra*, aminoglycosides *(aacC2*, *aadD*), quinolones (*flor*, *oqxA*), glycopeptides (vancomycin) *vanA*, tetracyclines *(tetC*, *tetO*), sulfonamides (*Sul1,Sul2, Sul3*), β- Lactams (*blaOXA-10*, *blaSHV*, *blaCTX-M, ampC*), efflux pump *qacE Δ 1*, Integron *int1* and transposon (*tnpA-IS4*, *tnpA-IS6*) and other antibiotic resistance genes. Considering the content and detection rate in the sample, we selected the above 11 antibiotic resistance genes with higher concentration for detection and analysis in this experiment.

### 4.4. Antibiotic Residue Analysis

Residues of sulfa antibiotic (sulfathiazole, sulfadiazine, sulfamethazine, sulfadimethoxine, sulfamethoxazole, and sulfisoxazole) in Min-xin river water were determined by field-amplified sample injection–CZE (FASI–CZE) coupled with a diode array detector, as previously reported [46]. The conditions for capillary electrophoresis analysis were as follows: the background electrolyte solution consisted of 70.0 mmol·L^−1^ borax and 60.0 mmol·L^−1^ boric acid (including 10% methanol, pH 9.1); the plug was 2.5 mmol·L^−1^ borax, which was injected into the capillary at a pressure of 0.5 psi for 5 s; then the sample was injected into the capillary at an injection voltage of −10 kV for 20 s; the electrophoretic separation was carried out under a voltage of +19 kV. The capillary temperature was maintained at 20 °C throughout the analysis, and six sulfonamides were completely separated within 35 min.

β-lactams residues (oxacillin, cloxacillin, dicloxacillin, and flucloxacillin) were extracted using polypyrrole-coated magnetic nanoparticles and subsequently analyzed by micellar electrokinetic capillary chromatography coupled with magnetic solid-phase extraction, as previously described [47]. In this experiment, Fe_3_O_4_ nanoparticles were prepared by chemical coprecipitation method. Polypyrrole was synthesized by polymerization of pyrrole monomer under the oxidation of ferric chloride and modified on Fe_3_O_4_ nanoparticles to obtain PPy/Fe_3_O_4_ nanoparticles. Then PPy/Fe_3_O_4_ was used for the extraction of sulfa antibiotic residues in water sample. 

Residues of 13 antibiotics from four categories of six sulphonamides, five β-lactams, one glycopeptides and chloramphenicol in soil along Min-xin river were determined by high performance capillary electrophoresis (HPCE) coupled with solid phase extraction (SPE) [48]. The data of these antibiotic residues were used as reference.

### 4.5. DNA Extraction

The frozen filter membrane was carefully removed from the centrifuge tube and then subjected to DNA extraction by an E.Z.N.A.TM Water DNA Kit. The extracted DNA was placed in a 1.5 mL EP tube and stored at −20 °C. After mixing, the soil sample (1.0 g) was placed in a 15-mL centrifuge tube, subjected to DNA extraction using an E.Z.N.A.TM soil DNA Kit, and then frozen stored in a 1.5-mL EP tube.

### 4.6. Fluorescence Quantitative PCR

In this experiment, tetracycline resistance genes *tetC* and *tetO*, sulfonamide resistance genes *sul1, sul2,* and *sul3,* macrolide resistance genes *ermA* and *ermB*, β-lactams resistance genes (*blaOXA-10*, *blaSHV*, *blaCTX-M*, *ampC*), class 1 integron-integrase gene *intI1*, transposon *tnpA-IS4*, and 16S rRNA internal reference gene were selected. Class 1 integrons are found widely in different environments and are the most studied integrons. The primers used for amplification and lengths of the targeted genes are listed in Table 4.

PCR assay was performed on a CFX96 real-time (RT) PCR detection system. In the fluorescence quantitative PCR detection, SYBR green dye was adopted. The total reaction volume was 25 μL. The reaction consisted of 12.5 μL of 2 × SYBR mix, 2 μL of upstream primer (10 μM), 2 μL of downstream primer (10 μM), 2 μL of template DNA, and 8.5 μL of ddH_2_O. The program is set as follows: pre-denaturation at 94 °C for 3 min, denaturation at 94 °C for 5 s, annealing at 57 °C for 15 s, and extension at 72 °C for 30 s, each for a total of 40 cycles. The concentration of the constructed plasmid containing the target gene fragment was serially diluted 10-fold. Then, the working curve of the sample was prepared using the Ct value measured by q-PCR. The temperature range of the melting curve was from 72 °C to 95 °C. The fluorescence signal was collected at each 0.5 °C increment and used for drawing the melting curve.

The sample and a known concentration of plasmid standard containing the same gene were simultaneously detected by qPCR, and the corresponding Ct value was obtained respectively. Then a gradient dilution of the plasmid concentration was performed to obtain a standard curve for quantification. Different resistance genes have different plasmid standards. Each ARG corresponds to a plasmid standard curve containing the same gene. After qPCR detection, the Ct value of ARG and plasmid standard series with known concentration was obtained, so as to quantify ARG.

Only the Ct value is obtained in the qPCR results. Through the standard curve of Ct value of the corresponding known plasmid concentration, the concentration multiple of the kit, the filtration volume of river water and the dry weight of soil, the absolute concentrations of various ARGs are finally obtained. Unit of absolute abundance is copies/g (dry mass of soil) or copies L (water sample), respectively.

### 4.7. Data Processing

Bio-Rad CFX Manager 3.1 software and EXCEL 2013 were used for data processing. One-way analysis of variance was used to analyze the differences between ARGs in water samples from different locations. Paired t-test was conducted to analyze the difference of ARGs in soil samples from two locations. Heatmap was utilized to visually compare the relative abundance of ARGs in different environmental samples. Pearson correlation was employed to analyze the correlation between ARGs and integrons and transposons.

## 5. Conclusions

In summary, real-time fluorescent quantitative PCR technique was used to detect antibiotic resistance genes in an urban river and soil near a veterinary antibiotic factory in Shijiazhuang, China. The results provided evidence that the relative abundance of ARGs in water and soil near the wastewater discharge outlet of the pharmaceutical factory was significantly higher compared to that in non-polluted areas, which is a proof that the sewage outlet discharged wastewater by an antibiotic manufacturing company, to a certain degree, is the source of pollution detected in the local environment. The most abundant ARGs detected in the study area environment were sulfa *sul1* and β-lactam *blaSHV.* Months of the year were also found to influence the variety of ARGs. Specifically, significant correlation between antibiotic resistance genes and mobile genetic elements was observed, and this was the likely factor that promoted the horizontal transfer of ARGs, allowing them to further spread. The possible resistance gene pollution to the local environment and the impact on microbial community and biodiversity caused by antibiotic production and their wastewater and waste gas emission in this region need to be further studied.

## Figures and Tables

**Table 1:** ANOVA analysis of ARGs at different sampling points at different times.

Relative Abundance	October		December		March	
	F *	*p* *	F	*p*	F	*p*
R*tetC*	0.264	0.850	1.983	0.163	0.304	0.822
R*tetO*	3.270	0.053	1.533	0.259	0.978	0.404
R*sul1*	2.843	0.071	34.329	0.000	1.922	0.167
R*sul2*	0.954	0.440	0.125	0.944	6.919	0.004
R*sul3*	0.747	0.548	1.727	0.215	2.985	0.261
R*ermA*	-	-	-	-	0.204	0.843
R*ermB*	3.920	0.073	6.595	0.008	0.508	0.683
R*blaSHV*	2.837	0.073	3.011	0.066	26.119	0.000
R*blaCTX-M*	3.483	0.058	-	-	37.296	0.000
R*blaOXA-10*	0.701	0.565	25.305	0.000	1.402	0.278
R*ampC*	3.854	0.030	13.569	0.000	14.981	0.000
R*int1*	0.314	0.815	3.512	0.049	4.720	0.015
R*tnpA IS4*	0.550	0.656	26.040	0.000	2.637	0.090

Notes *: F: F value of ANOVA; *p*: The correlation compared with 0.05.

**Table 2:** The value of Pearson correlation between ARGs and *int1* and *tnpA IS4* at different times.

Relative Abundance	October		December		March	
	R*int1*	R*tnpA IS4*	R*int1*	R*tnpA IS4*	R*int1*	R*tnpA IS4*
R*tetC*	0.74 *	0.37	−0.56 *	0.14	0.18	0.27
R*tetO*	0.56 *	−0.23	0.57 *	0.47	0.35	0.22
R*sul1*	0.59 *	−0.33	−0.21	0.95 *	0.74 *	0.68
R*sul2*	0.01	0.21	0.06	0.14	−0.37	−0.12
R*sul3*	0.02	0.23	0.32	−0.3	−0.01	0.26
R*ermA*	-	-	-	-	−0.94	−0.73
R*ermB*	0.35	0.71	0.59 *	0.87 *	0.56 *	0.42
R*ampC*	−0.19	0.62 *	0.47	−0.84 *	−0.45	0.59 *
R*blaSHV*	−0.3	0.51 *	−0.04	−0.65 *	−0.50 *	−0.18
R*blaCTX-M*	0.24	0.37	-	-	−0.5	0.62
R*blaOXA-10*	0.44	0.57 *	0.6 0 *	0.83 *	0.44	0.25

Notes: * Correlation is significant at the 0.05 level (2-tailed).

**Table 3:** Soil moisture content (%).

Sample	October		December		March	
	Point A	Point B	Point A	Point B	Point A	Point B
1	3.33%	-	1.67%	10.33%	2.38%	4.41%
2	3.38%	4.65%	4.00%	13.91%	1.67%	5.00%
3	1.67%	6.00%	4.20%	15.09%	2.98%	3.31%
4	2.00%	5.33%	14.92%	14.69%	1.33%	11.33%
5	4.00%	10.67%	4.33%	-	1.99%	1.99%

**Table 4:** PCR primers used for amplification of ARGs, class 1 integron, and transposon.

Types	ARGs	Primer Sequence	Amplicon Size (bp)	Reference
Tetracyclines	*tetC*	CATATCGCAATACATGCGAAAAA AAAGCCGCGGTAAATAGCAA	78	[50]
	*tetO*	ACGGAGAGTTTATTGTATACC TGGCGTATCTATAATGTTGAC	171	[50]
Sulfonamides	*Sul* *1*	CGCACCGGAAACATCGCTGCAC TGAAGTTCCGCCGCAAGGCTCG	162	[51]
	*Sul2*	TCCGGTGGAGGCCGGTATCTGG CGGGAATGCCATCTGCCTTGAG	190	[52]
	*Sul3*	TCCGTTCAGCGAATTGGTGCAG TTCGTTCACGCTTTACACCAGC	127	[52]
Macrolides	*ermA*	GAAATCGGATCAGGAAAAGG AACAGCAAACCCAAAGCTC	332	[53]
	*ermB*	GATACCGTTTACGAAATTGG GAATCGAGACTTGAGTGTGC	364	[53]
β-lactams	*blaOXA-10*	CGCAATTATCGGCCTAGAAACT TTGGCTTTCCGTCCCATTT	71	[54]
	*blaSHV*	GCGAAAGCCAGCTGTCGGGC ATTGGCGGCGCTGTTATCGC	302	[55]
	*blaCTX-M*	GGAGGCGTGACGGCTTTT TTCAGTGCGATCCAGACGAA	63	[56]
	*ampC*	CAGCCGCTGATGAAAAAATATG CAGCGAGCCCACTTCGA	147	[57]
Integron	*intI*	TCGTGCGTCGCCATCACA GCTTGTTCTACGGCACGTTTGA	67	[58]
Transposon	*tnpA-IS4*	GGGCGGGTCGATTGAAA GTGGGCGGGATCTGCTT	108	[54]
16S rRNA	*V4*	GTGCCAGCAGCCGCGGTAA GGACTACAAGGGTATCTAAT	292	[59]

## Data Availability

Not applicable.
